# Modelling levels of nitrous oxide exposure for healthcare professionals during EMONO usage

**DOI:** 10.1186/s40557-016-0116-1

**Published:** 2016-07-07

**Authors:** Marine Pichelin, Catherine Billoet, Georges Caillibotte

**Affiliations:** Air Liquide Santé International, WBL Healthcare, Medical R&D, 1 chemin de la Porte des Loges, 78350 Les Loges-En-Josas, France; Air Liquide Santé International, WBL Healthcare, Medical R&D, 28 Rue d’Arcueil, 94250 Gentilly, France

**Keywords:** 3D numerical simulation, Equimolar mix of nitrous oxide (N_2_O)/oxygen (O_2_), Occupational exposure limit

## Abstract

**Background:**

Computational fluid dynamics (CFD) has been used to compute nitrous oxide (N_2_O) levels within a room during the administration of an equimolar mix of N_2_O/oxygen (EMONO) in the clinical setting. This study modelled realistic scenarios of EMONO usage in hospital or primary care, in order to estimate the potential N_2_O exposure of healthcare professionals (HCP) with routine EMONO use and to provide guidance for EMONO users.

**Methods:**

Sixteen scenarios were defined by carrying out a survey of practitioners. CFD simulations were performed for each scenario and N_2_O concentrations over time were calculated. N_2_O exposures (time-weighted average of concentration over 8 h [TWA-8 h]) were calculated at the HCPs’ mouth to be compared with a predefined occupational exposure limit (OEL).

**Results:**

Administration duration and ventilation type were the main factors influencing N_2_O levels; ventilation type also influenced wash-out time between EMONO administrations. N_2_O concentration showed a plume distribution towards the ceiling and was highly heterogeneous, highlighting the importance of measurement location. Although estimated TWA-8 h varied widely, 13 of the 16 scenarios had an N_2_O TWA-8 h of <100 parts per million.

**Conclusions:**

Data demonstrate that EMONO usage in well ventilated rooms – as recommended – helps to ensure that N_2_O exposure does not exceed the OEL and does not signal any major risks for HCPs when recommendations are followed. Although these data are numerical simulations and should be considered as such, they can provide guidance for EMONO users.

## Background

The N_2_O/O_2_ 50 %/50 % admixture, also known as Equimolar Mixture of Oxygen and Nitrous Oxide (EMONO, Kalinox™ Air Liquide Santé International, France, Equanox™, Alnox™, Nitronox™), is indicated for the treatment of short-term pain conditions of mild to moderate intensity when rapid-onset powerful short-term relief of pain is required [1, 2]. The risks associated with chronic occupational N_2_O exposure for healthcare professionals (HCP) are still controversial [3–5]. Hence, for several decades, some cases of repeated and prolonged exposure have been reported to be linked to medical conditions including reproductive, neurologic, hepatic, and renal disorders [5, 6].

The National Institute for Occupational Safety and Health (NIOSH) recommended exposure limit (REL) is 25 parts per million (ppm) as a time-weighted average (TWA) in operating room during the period of general anesthesia and the American Conference of Governmental Industrial Hygienists (ACGIH) threshold limit value (TLV) for N_2_O is 50 ppm as an 8-h TWA (TWA-8 h) [7]. For HCPs administrating EMONO, specific occupational exposure limits (OELs) have been defined in some countries, for example in the US, the N_2_O OEL TWA-8 h is 50 ppm (90 mg/m^3^) [7] and in the UK and some European countries it is 100 ppm (180 mg/m^3^) [8–10].

These data are not easily measurable or predictable and experimental airflow studies are difficult and expensive due to their complexity [11]; therefore, Computational Fluid Dynamics (CFD) software is a useful tool that can be used to generate 3-dimensional (3D) numerical simulations to predict gas flow and gas concentrations in specified enclosed environments. It has been used in a wide range of applications such as investigating ventilation effectiveness in clinical settings such as operating rooms [11–18] and birthing rooms [19]. In other healthcare applications, CFD has been used successfully to assess the benefits of a laminar flow screen to reduce bacterial contamination in hospitals [11], and to investigate airborne contaminants in medical [18, 20–24] and dental clinics [25]. Other medical applications of CFD encompass analysis of blood flow through arteries [26, 27], assessing rupture risk in cerebral aneurysms [28], and analysis of exhaled respiratory gases [29, 30].

Ideally, adequate ventilation systems, room design, equipment maintenance, and clinical practices should ensure that staff are never subjected to excessive levels of N_2_O OEL [5]. In view of the difficulties and cost of measuring N_2_O distribution in real-life situations, the feasibility of using 3D numerical simulation with CFD software to predict the distribution of N_2_O concentrations with EMONO use within a room has been assessed. The comparison of computed data with experimental measures of N_2_O concentration within a laboratory showed good agreement, both in terms of maximal concentrations achieved during EMONO administration and in terms of the estimated TWA-8 h. The changes in N_2_O concentration over time during EMONO administration and wash-out phases were also correctly described in the simulation, supporting the relevance of using 3D numerical simulation to compute the N_2_O TWA-8 h for HCPs.

Hence, the objective of our work was to use a numerical modelling approach to compute the N_2_O concentration distribution in a room during various realistic scenarios of EMONO administration, to predict the associated TWA-8 h ppm N_2_O exposure for a range of HCPs using EMONO and assess the pertinence of the current EMONO usage recommendations.

## Methods

Scenarios and parameters used: In order to perform CFD simulation, detailed modelling of the room features such as dimensions, patient’s location, ventilation, door and window, the initial conditions within the room, and various input parameters such as EMONO flow rate and administration time are required. As an infinite number of scenarios are possible, for practical reasons, we defined 16 scenarios most representative of real life EMONO administration by surveying various HCPs using EMONO for different indications, such as painful procedures in children and adults emergency rooms, or for dermatology, dentistry, and obstetrics procedures.

For each scenario, a full set of environmental and EMONO administration parameters was specified. Environmental parameters included room size, the type of ventilation present (natural via windows and doors or a controlled mechanical ventilation system or extraction system), the number of subjects located within the room in addition to the HCP (an adult patient or a child patient and his/her parent), the temperature of the room (and outside temperature if windows and doors were open), the number of windows and doors and the location of the air inlet/outlet. The EMONO administration parameters included EMONO flow rate and duration, continuous or intermittent administration mode, the use of an on-demand valve or not, and the number of successive repetitions of EMONO administrations (Table 1).

**Table 1:** The 16 scenarios used to represent real-life equimolar mix of nitrous oxide (N2O)/oxygen (O2) [EMONO] administration

Scenario	Patient	Room size (m^2^)	Room ventilation mode	Administration mode	EMONO usage	TWA-8 h ppm (OEL = 25–100)
#1 Dentistry	Adult	15	Controlled mechanical ventilation system (5 vol/h)	Without on-demand valve	(20 min adm. + 40 min washout^a^) ×2	**19.0**
#2 Dentistry					(59 min adm. + 40 min washout^a^) ×1	28.7
#3 Dentistry			Window		(20 min adm. + 40 min washout^a^) ×2	**20.4**
#4 Dentistry					(59 min adm. + 40 min washout^a^) ×1	65.6
#5 Dermatology	Adult	10	Single door	On-demand valve	(30 min adm. + 30 min washout^a^) ×2	57.2
#6 Emergency room	Child (+ parent)	10	Double door	Without on-demand valve	(15 min adm. + 20 min washout^a^) ×3	**8.7**
#7 Emergency room					(59 min adm. + 20 min washout^a^) ×3	129.6
#8 Emergency room				On-demand valve	(15 min adm. + 20 min washout^a^) ×3	**4.2**
#9 Emergency room					(59 min adm. + 20 min washout^a^) ×3	64.2
#10 Emergency room	Adult	10	Double door	Without on-demand valve	(15 min adm. + 20 min washout^a^) ×3	48.3
#11 Emergency room					(59 min adm. + 20 min washout^a^) ×3	371.4
#12 Emergency room				On-demand valve	(15 min adm. + 20 min washout^a^) ×3	**21.9**
#13 Emergency room					(59 min adm. + 20 min washout^a^) ×3	175.5
#14 Obstetrics	Adult	15	Extraction system (15 vol/h)	On-demand valve Intermittent inhalation^b^	(59 min adm. + 40 min washout^a^) ×1	**2.0**
#15 Radiology	Adult	15	Single door	On-demand valve	(10 min adm. + 30 min washout^a^) ×1	**4.6**
#16 Radiology					(30 min adm. + 30 min washout^a^) ×1	**23.7**

Each scenario is defined by an associated real-life situation (e.g., dentistry, emergency room, etc.), the patient type (adult or child), the room size, the room ventilation mode (natural ventilation such as window or door opening, or mechanical ventilation system with a change volume rate of 5 vol/h or 15 vol/h), the administration mode (with or without on-demand valve), and the EMONO usage (i.e., administration duration plus computed wash-out time, and number of successive usages). The TWA-8 h ppm values computed at the HCP’s mouth are also presented. Bold values are below the most restrictive OEL of 25 TWA-8 h ppm. Note that the computed TWA-8 h ppm values are above the less restrictive OEL (100 ppm) for three scenarios only. ^a^See the text for a definition of the wash-out time. ^b^Intermittent inhalation corresponds to successive periods of 3 min with EMONO administration and 3 min without EMONO administration

**Fig. 1:**
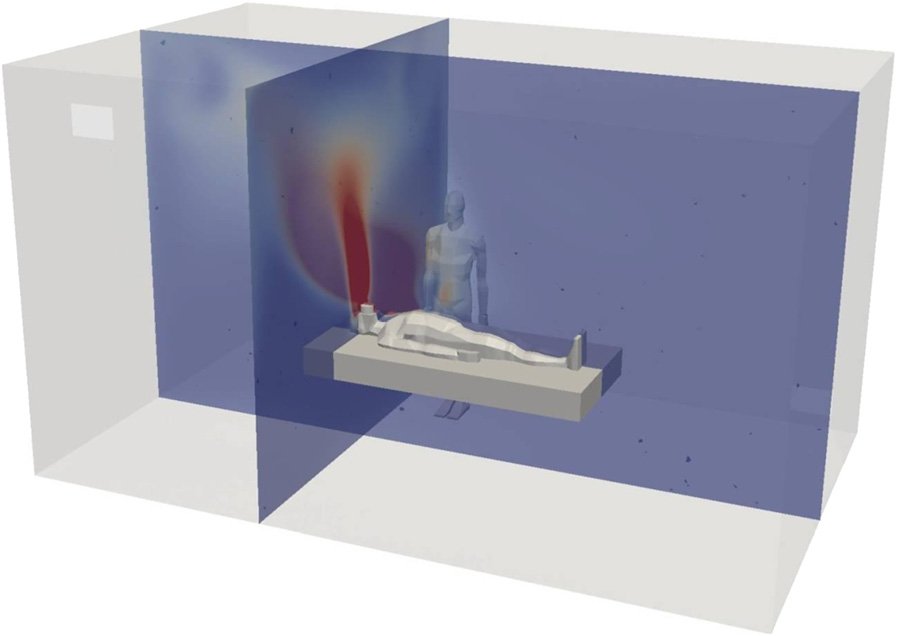
Virtual representation of a medical room, including an adult patient in supine position on a bed at the center of the room and the healthcare professional standing close to the patient’s bed. In addition are represented in two 2D-plane sections the N_2_O concentration values within the room, as obtained by the numerical simulation, at one given time during the equimolar mix of nitrous oxide (N_2_O)/oxygen (O_2_) [EMONO] administration process, as defined in one of the considered scenarios. The red and dark blue colors are representative of the maximal and minimal computed concentrations, respectively

Numerical simulations of these 16 scenarios based on real conditions of EMONO in hospital or in primary care setting were performed using CFD software. A virtual representation of a room was modelled in 3D (Fig. 1) in accordance with each scenario, using seven geometries with variations in room size, presence of window, single or double doors, mechanical ventilation systems, child or adult patient (and presence of parent): two different geometries for dentistry, one for dermatology, two for emergency treatment rooms (child and adult), one for obstetrics and one for radiology.

The patient was represented as having a supine position on a bed at the center of the room, and the HCP was represented as standing close to the patient’s bed. The parent, if featured in the scenario, was represented as standing close to the child patient on the opposite side from the HCP.

Human bodies (HCP + patient + parent if child patient) were represented as being realistic human body shapes (Fig. 1). Indeed, the shape of the body has been shown to affect gas distribution and concentrations around a human body with a rounded realistic body shape compared to oversimplified geometrical shape to represent the body [22, 24]. Any motion of practitioners or parent was not included in the model.

A leak was assumed to exist at the mask level – defined as a cylindrical surface 10 cm in diameter and 1 mm in width at the edge of the mask – and was the source of N_2_O presence in the room. The leak was assumed to be 10 % of the EMONO flow rate (i.e. 10 % of 6 L/min for a child and 10 % of 12 L/min for an adult patient). The species model assumed 0.79 N_2_ + 0.21 O_2_ mole fractions in the room initially and the leak at the mask level was defined to be 0.5 N_2_O + 0.5 O_2_ mole fractions. For scenarios using continuous EMONO administration (i.e., without on-demand valve), the leak was assumed to be continuous, whereas with an on-demand valve, the leak was assumed to occur only during the inspiration phase.

Turbulence modelling was included, as well as appropriate models to take into account thermal effects, e.g. temperature of the walls and room, and natural convection due to heat flux from the bodies. Turbulence has been observed to be an important phenomenon for accuracy of CFD modelling and for the optimum location of sampling points [14, 22, 24, 31–33]. The temperature of the room and EMONO were included in the model. The temperature of primary care offices (dentistry and dermatology) was assumed to be 20 °C with corridor 18 °C and outside 15 °C, and a hospital room (emergency, radiology, and obstetrics) was assumed to be 23 °C, with corridor 21 °C. The temperature of EMONO was assumed to be equal to the ambient room temperature. To model the heat released by people present in the room (and model natural convection), the following values were used: supine child patient = 55 W (heat flux Q = 74 W/m^2^); supine adult patient = 80 W (Q = 65.5 W/m^2^); relaxed parent = 110 W (Q = 63.7 W/m^2^); and working medical staff = 143 W (Q = 82.6 W/m^2^). Since the air initially present in the room and the EMONO administered to the patient can be considered as incompressible gases, the volume of gas entering the room due to EMONO inflow has to be equal to the volume of gas leaving the room to ensure mass conservation. Therefore, in the model an outflow surface at the bottom of the door (a door leak) was included to permit some air flow even when closed.

Fluent CFD software (ANSYS, PA, USA) was used to compute the N_2_O concentration in each part of the room (millions of points), at every second of the administration process, for each scenario. Because the location of the measurement point is known to influence the TWA-8 h value [22, 33, 34], a virtual sensor (probe) was positioned by the HCP’s mouth, to be representative of the inhaled exposure. The N_2_O exposure level for the HCP (TWA-8 h ppm) was computed using the simulated values of N_2_O concentration at this probe throughout the simulated scenario.

A wash-out phase was included in each scenario, following the EMONO administration phase. The wash-out time is defined as the time needed to evacuate at least 95 % of the N_2_O mass present in the room at the end of the EMONO administration phase. When there is no mechanical ventilation system in place, the (single or double) door or window is supposed to be closed during administration and open during the wash-out phase. Hence, when this natural ventilation starts, the N_2_O concentration within the room starts decreasing. For scenarios considering an active ventilation system in place, this system is supposed to run during both administration and wash-out phases.

Data on N_2_O concentration (ppm), temperature (°C), and gas velocity (m/s) were gathered for each scenario throughout the EMONO administration and wash-out phases. Quantitative data were visualized in 2D and 3D; an example of the computed N_2_O concentration values represented in two 2D slices intersecting the mask for one scenario is shown in Fig. 1.

**Fig. 2:**
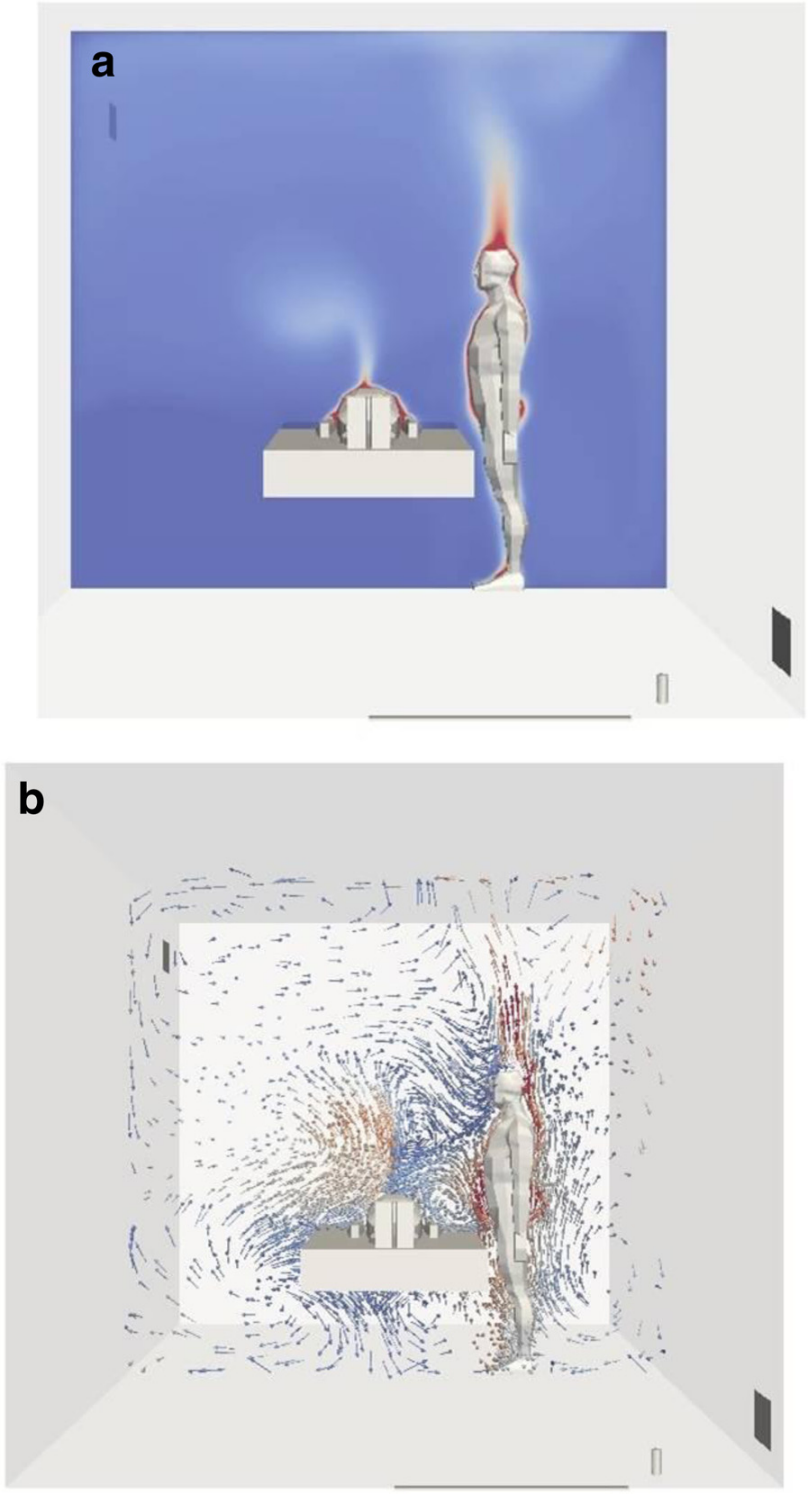
**a** Representation of the computed temperature within the room in a 2D slice. The red and dark blue colors are representative of the maximal and minimal temperatures, respectively. One can note the higher temperature near the body shapes, highlighting the presence of the heat flux released by human subjects. **b** Representation of the gas motion in a 2D slice. The arrows indicate the local direction of the gas mixture and are colored by the local gas mixture velocity (the red and dark blue colors are representative of the maximal and minimal velocity magnitudes, respectively). The influence of the natural convection is clearly shown by the upward direction of the gas mixture above the healthcare professional, where the temperature is the highest. Note that these figures are the results of one of the considered scenarios, but are representative of all the scenarios

Computed temperature and gas motion and velocity within the room were modelled to demonstrate the influence of natural convection (Fig. 2).

As the N_2_O concentration is numerically computed throughout the room and over time, the N_2_O concentration at the HCP’s mouth was calculated at each second of the administration and wash-out phases. The N_2_O exposure in TWA-8 h ppm at the HCP’s mouth probe was calculated for each scenario to be compared with country-dependent OELs. In addition, the evolution of N_2_O TWA-8 h ppm over time can be computed and gives useful insight on exposure levels.

## Results

N_2_O did not accumulate at floor level, instead the N_2_O concentration tends to show a plume distribution, orientated toward the ceiling (Figs. 1 and 2). However, the global N_2_O concentration distribution is very different for the various scenarios tested (affected by duration, use of mechanical ventilation, on-demand valve, etc.).

**Fig. 3:**
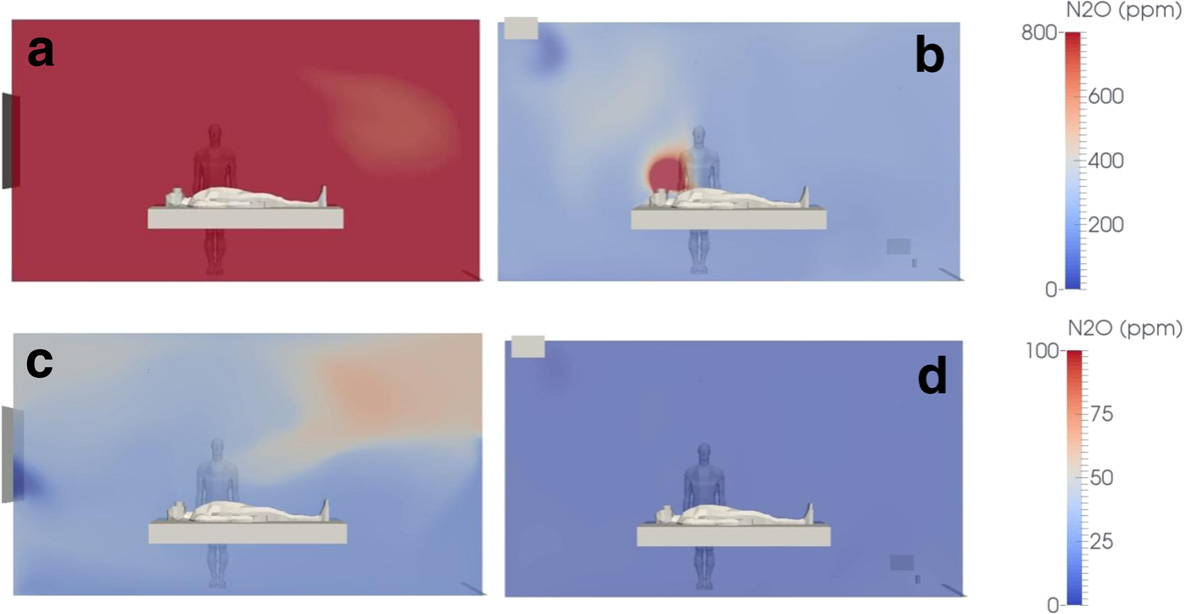
Nitrous oxide (N_2_O) concentration distribution in ppm at a single 2D plane located along the patient’s bed at the end of a 59 min EMONO administration phase (**a** and **b**) without any ventilation apart from window opening (**a**: case #4) and with mechanical ventilation of 5 vol/h (**b**: case #2). Data are also shown in lower panels (**c** and **d**) for the same scenarios at the end of a subsequent 40 min wash-out phase (**c**: window opening; **d**: mechanical ventilation 5 vol/h). The red and dark blue colors are representative of the maximal and minimal computed concentrations, respectively. Note the different scale for color depiction of N_2_O concentration in lower panels

**Fig. 4:**
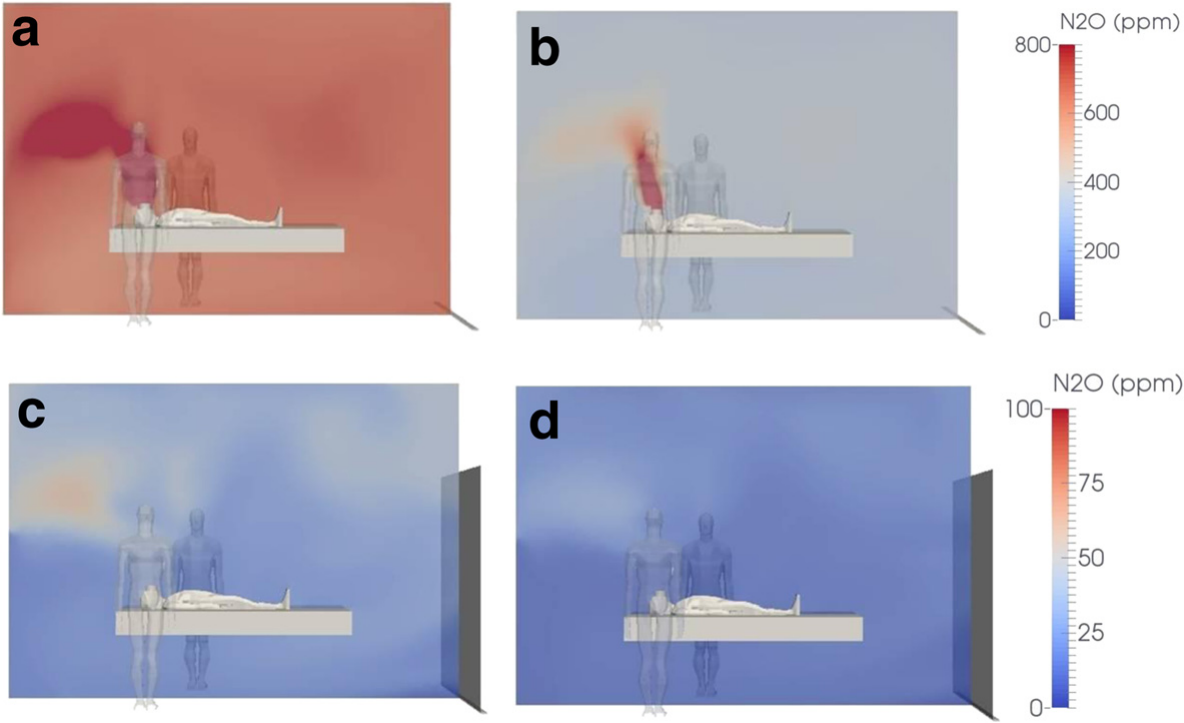
Nitrous oxide (N_2_O) concentration distribution in ppm at a single 2D plane located along the patient’s bed (pediatric patient) at the end of a 59 min EMONO administration phase (**a** and **b**) without on-demand valve (**a**: case #7) and with on-demand valve (**b**: case #9). Data are also shown in lower panels (**c** and **d**) for the same scenarios at the end of a subsequent 15 min wash-out phase (**c**: case #6; **d**: case #8; double-door opening in both cases). The red and dark blue colors are representative of the maximal and minimal computed concentrations, respectively. Note the different scale for color depiction of N_2_O concentration in lower panels

The change in N_2_O concentration in the room calculated for each scenario showed a clear influence of the specific ventilation system used. For example, in the dental treatment room, the N_2_O concentration level was higher without an active ventilation system, particularly with longer administration durations (Fig. 3). With active ventilation, a plateau effect is induced over time, at which point, the N_2_O global concentration in the room remains constant to the end of the current EMONO administration. Similarly, administration without an on-demand valve resulted in considerably higher N_2_O concentrations compared with administration using an on-demand valve, particularly with longer administration durations (Fig. 4).

It has to be noted that computed wash out times varied from 20 to 40 min, highlighting the role of a delay between two EMONO administrations. The decrease in N_2_O concentration during the wash-out phase was quicker when a double-door was open (compared with a single door or window), but is highly dependent on the temperature gradients used in the model. Therefore, the duration of the wash-out phase would be different if other temperatures were selected for the model.

**Fig. 5:**
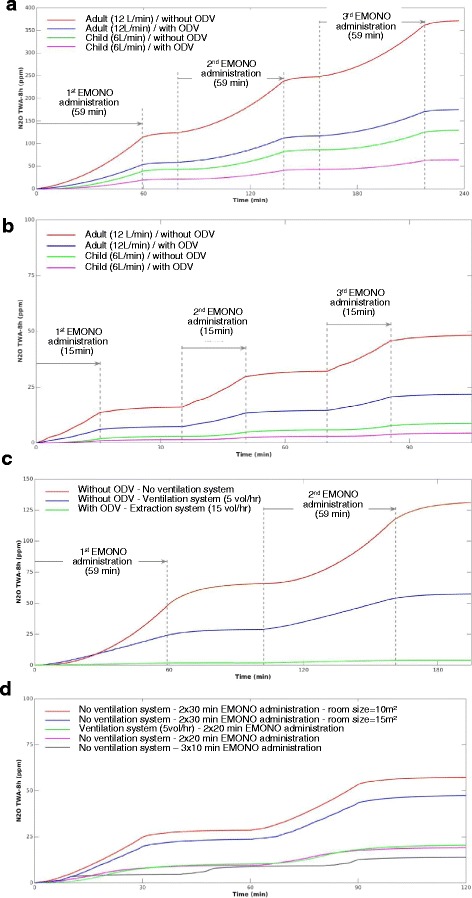
Change in the time-weighted average of nitrous oxide (N_2_O) concentration over an 8 h period (TWA-8 h) values over time for various scenarios. In Panels (**a** and **b**), the room is considered non-ventilated, except for a door opening between successive administrations of equimolar mix of nitrous oxide (N_2_O)/oxygen (O_2_) [EMONO]. In Panels (**c** and **d**), only adult patients are considered and a door or window opening is simulated between successive EMONO administrations for the otherwise non-ventilated rooms. ODV stands for on-demand valve

The estimated TWA-8 h values varied widely (from 2 to 371 ppm), illustrating the influence of the conditions of EMONO administration. Thirteen of the 16 scenarios had TWA-8 h <100 ppm, including eight scenarios with TWA-8 h <25 ppm. The changes in TWA-8 h values over time obtained by numerical simulations were computed (Fig. 5). The influence of the administration duration is clearly shown in panels A and B, whereas the importance of the ventilation system is highlighted in panel C. Shorter procedures are presented in panel D.

## Discussion

Our computed numerical results of N_2_O levels in common clinical situations tend to demonstrate that administration of EMONO in well ventilated rooms, and/or with on-demand valve, and/or for short duration procedures, as recommended, would ensure that the N_2_O exposure remains below the OEL. These findings do not signal any major risks for the HCP when these recommendations are followed.

The simulated data show very high N_2_O concentration near the mask and considerable differences among the various scenarios tested, as expected. The data also show substantial heterogeneity in N_2_O concentration throughout the room in many scenarios, highlighting the importance of the location of the probe used to determine exposure and showing that using one point of measurement may not be representative of HCP N_2_O exposure.

Importantly, N_2_O does not accumulate at floor level, as might be expected due to the higher density of N_2_O compared with that of air, instead the N_2_O concentration tends to show a plume distribution, oriented toward the ceiling. Plume formation is due to the uneven temperature distribution throughout the room inducing movement of gases via natural convection. The temperature gradients observed in the room are mainly due to the heat generated by the human bodies (HCP, patient, and parent). Numerical simulation with CFD has been used to predict heat release from a human body using modelling of airflow and thermal radiation [14, 35] and CFD has also shown that using realistic human shapes is important in accurate modelling of thermal radiation and impact on air flow and turbulence [31].

Our data also demonstrate the importance of the recommended wash-out phase between successive N_2_O administrations to minimize the overall cumulative N_2_O exposure over time. Wash-out times were similar for children and adults, with and without an on-demand valve, and with and without active ventilation.

In this study, it was calculated that the N_2_O TWA-8 h was >100 ppm (i.e. higher than the most relaxed OEL) at the HCP’s mouth in only three scenarios – all of which were related to using a long EMONO administration (at least 59 min) with no active ventilation system. This highlights the importance of active ventilation particularly for longer durations of EMONO use.

Limitations of this study are those inherent to any model in that the data are hypothetical and only pertain to the 16 scenarios tested (e.g. size of the window, outside temperature). Beyond the influences on N_2_O concentration distribution described herein, there are several others that are beyond the scope of this study. For example, the motion of the practitioners is not included in the simulations (i.e., the HCP stays in the same room and same position for eight hours). This is unlikely in real life situations, but using this hypothesis leads to an overestimation of the theoretical N_2_O exposure of the HCP. However, in terms of CFD, computational aspects of the simulations have been checked for accuracy using standard practices in the field.

## Conclusion

In conclusion, the current EMONO usage recommendations state that the product should be used in airy, well ventilated rooms. Our simulated data demonstrate that following these recommendations, together with use of an on-demand valve and/or for short procedures, would ensure that the N_2_O exposure for HCPs does not exceed the TWA-8 h OEL defined for each country. It should be noted that these results are derived purely from numerical simulations and should be considered as such; however, earlier research has shown the validity of using CFD to simulate gas levels in treatment rooms and so they can be considered a good estimation of N_2_O levels likely with EMONO used in the scenarios studied.

It is hoped that these data will provide useful information and guidance for EMONO users to ensure exposure recommendations are followed.

## Abbreviations

3D, 3-dimensional; ACGIH, American conference of governmental industrial hygienists; CFD, computational fluid dynamics; EMONO, equimolar mix of N2O/oxygen; HCP, healthcare professionals; N_2_O, nitrous oxide; ODV, on-demand valve; OEL, occupational exposure limit; ppm, parts per million; TWA-8 h, time-weighted average of concentration over 8 h.
